# End-of-life circumstances and unanticipated deaths in a neonatal intensive care unit: a retrospective analysis

**DOI:** 10.1186/s12904-026-02014-2

**Published:** 2026-02-23

**Authors:** Clara Perenyi, Gilles Cambonie, Sabine Durand, Florence Vachiery Lahaye, Arthur Gaudaire, Christophe Milesi, Arthur Gavotto

**Affiliations:** 1 Department of Neonatal Medicine and Pediatric Intensive Care, Arnaud de Villeneuve Hospital, Montpellier University Hospital Centre, 371 Avenue du Doyen Giraud, Montpellier, 34295 France; 2 Department of Neurosurgery, Gui de Chauliac Hospital, and Donation and Transplantation Coordination Unit, Montpellier University Medical center, Montpellier, 34090 France; 3 PhyMedExp, CNRS, INSERM, University of Montpellier, 371 Avenue du Doyen Giraud, Montpellier, 34295 France

**Keywords:** Neonatal intensive care, End-of-life care, Withholding and withdrawal of life-sustaining treatment, Palliative care, Anticipated and unanticipated deaths, Ethics, Parental support

## Abstract

**Background:**

Despite major advances in neonatal intensive care, mortality in neonatal intensive care units (NICUs) remains a persistent reality. Most deaths now occur after withholding or withdrawing life-sustaining therapies (WWLST), yet some remain sudden or unanticipated. Understanding how and under which circumstances infants die is essential to improving anticipatory communication, ethical consistency, and family-centered support in neonatal end-of-life (EOL) care.

**Methods:**

We conducted a retrospective study including all infants who died in the tertiary NICU of Montpellier University Hospital, France, between May 2022 and May 2025. Demographic, perinatal, clinical, and end-of-life data were extracted from medical records. Deaths were classified as anticipated (following WWLST) or unanticipated (without WWLST). Statistical comparisons explored factors associated with unanticipated deaths.

**Results:**

Among 870 NICU admissions, 105 infants (12%) died. Mortality was concentrated in three groups: very premature infants (< 29 weeks’ gestation, 55%), infants with severe congenital or early-onset conditions (26%), and those with hypoxic-ischemic encephalopathy (19%).

Seventy-six infants (72%) died after a WWLST decision—most often for poor neurological prognosis or perceived futility of care—whereas 23 (22%) died without a prior WWLST decision. Unanticipated deaths were mainly associated with multi-organ failure (≥ 3 organs 65% vs 40%; *p*=0.02), predominantly cardiovascular or respiratory failure, shorter illness trajectories (1 vs 6.5 days from complication to death; *p*<0.01), and reduced parental presence during EOL care (52% vs 80%; *p*<0.01), including fewer opportunities for parents to be present and to hold their infant at the time of death.

**Conclusions:**

In this tertiary NICU, most deaths were anticipated and occurred following structured WWLST processes. Unanticipated deaths primarily reflected rapid clinical deterioration and were associated with more abrupt and less family-centered end-of-life circumstances. These findings underscore the importance of early recognition of dying trajectories and timely multidisciplinary discussions to support anticipatory, compassionate, and parent-centered end-of-life care in the NICU. Strengthening education in neonatal ethics and palliative care may foster more consistent, compassionate, and anticipatory EOL practices.

**Supplementary Information:**

The online version contains supplementary material available at 10.1186/s12904-026-02014-2.

## Background

Despite major advances in neonatal intensive care in high-income countries - such as improved ventilation, infection control, and neuroprotective strategies [1–3] - mortality in neonatal intensive care units (NICUs) remains a persistent reality, accounting for a substantial proportion of overall infant deaths. According to the World Health Organization, in 2022, 47% of all deaths among children under five occurred during the neonatal period [4]. In France, neonatal mortality has shown an unfavorable trend over the past decade. Following a steady decline from 2001 to 2012, the national neonatal mortality rate subsequently increased, reaching 3.63 per 1,000 live births, largely due to a rise in early neonatal deaths (days 0–6) [5]. In high-income countries, most neonatal deaths occur following end-of-life (EOL) decisions, particularly the withholding or withdrawal of life-sustaining therapies (WWLST) [6]. EOL care in neonatology is an emotionally intense and ethically complex experience for both parents [7] and healthcare professionals [8]. Providing compassionate, structured, and family-centered support is therefore a cornerstone of neonatal palliative care, with reported WWLST rates ranging from 38% to over 90% [6, 9–11], reflecting variations in patient populations, cultural values, and ethical or legal frameworks.

However, prognostic uncertainty, particularly in very preterm infants and those with severe neurological injury [12], often complicates decision-making. Considerable heterogeneity also persists in communication with families, ethical consultation, and integration of palliative-care principles [13], while formal training in these areas remains limited [14].

Understanding how infants die and under which circumstances is essential to improving the quality, consistency, and humanity of neonatal EOL care. This study aimed to describe who dies and how in our NICU, and to identify modifiable factors associated with unanticipated deaths, in order to strengthen anticipatory and supportive-care strategies.

## Methods

### Study design and population

This retrospective, single-center study was conducted in the NICU of Montpellier University Hospital, France — a 14-bed tertiary care unit — and included all eligible cases between May 2022 and May 2025. Eligible participants were liveborn infants who died within the NICU during the study period. Infants who died in the delivery room or after being transferred to another department for end-of-life care were excluded.

### Framework for withholding or withdrawing life-sustaining therapies (WWLST) in France

Ethical decisions in neonatal care are guided by the principles of beneficence, nonmaleficence, respect for parental autonomy, and justice. In France, end-of-life practices are regulated by the Claeys–Leonetti law (2016), which defines patients’ rights regarding care considered futile or disproportionate. This law establishes three major principles: (i) the use of advance directives when available, (ii) the right to deep and continuous sedation until death when WWLST is indicated, (iii) and the requirement for a collegial decision-making process involving the medical and nursing teams and, whenever possible, the family.

In neonatal intensive care, these ethical and legal principles raise specific challenges, as infants cannot express their wishes. Decisions are therefore shared between the medical team and the family, based on prognosis, potential suffering, and the best interests of the child.

For the purpose of this study, WWLST decisions were classified as follows: (i) Types 1 and 2 WWLST defined as maintaining current therapies without introducing new interventions (type 1) or without increasing the intensity of ongoing support (type 2); (ii) Type 3 WWLST: withdrawal of ongoing life-sustaining therapies (e.g., mechanical ventilation, ExtraCorporeal Membrane Oxygenation [ECMO], renal replacement therapy, inotropes, artificial nutrition or hydration); (iii) Emergency WWLST: urgent decisions made outside standard hours that may involve either Type 1 and 2 or Type 3 situations, subsequently reviewed within a collegial framework.

### Data collection

Data were extracted from electronic medical records. The following variables were recorded: (i)Perinatal and demographic data: sex, birth weight, gestational age, place of birth (inborn/outborn), antenatal diagnosis, and Apgar scores at 1, 5, and 10 min; (ii) Clinical course and timing: age at death; interval between admission and the major complication (defined as the event significantly altering the clinical course); time between complication and death; time between WWLST decision and death; and use of renal replacement therapy (peritoneal or hemodialysis); (iii) Organ failure before death: presence of organ failures immediately preceding death, classified according to the Pediatric Organ Dysfunction Information Update Mandate (PODIUM) criteria [15]; (iv) End-of-life conditions: anticipated (following WWLST) or unanticipated deaths; type of WWLST (Type 2 or 3); mode of death (planned extubation, deep and continuous sedation, or non-recovered cardiac arrest); parental presence at the time of death; and whether the infant was held in the parents’ arms during the dying process.

Patients were categorized into three main clinical groups commonly observed in neonatal intensive care: (i) very preterm infants (< 29 weeks’ gestation age [GA]); (ii) term infants with hypoxic-ischemic encephalopathy; and (iii) infants with severe congenital or early-onset conditions (including congenital malformations, genetic syndromes, and early postnatal organ failure). We deliberately used descriptive clinical terminology to distinguish between structural congenital malformations and early-onset organ failure, rather than disability-based labels, in order to improve international clarity and respect for families.

Data extraction was performed by two investigators, with cross-checking of all records to ensure consistency and accuracy.

### Outcome

The primary outcome was to describe the incidence and characteristics of infants who died in the NICU during the study period, including demographic, perinatal, clinical, and EOL factors.

The secondary objective was to analyze the circumstances surrounding deaths that occurred without a prior WWLST decision—defined as unanticipated deaths—and to identify clinical or organizational factors that could help distinguish these cases from anticipated deaths following a WWLST process. This distinction aimed to better understand potentially modifiable elements of end-of-life care and to strengthen anticipatory and supportive-care strategies within the NICU.

### Ethical considerations

This study was approved by the Institutional Review Board of Montpellier University Hospital (CSE-2025-07-305). It was conducted in accordance with the ethical standards of the institutional and national research committees and with the 1964 Declaration of Helsinki and its later amendments. In compliance with French legislation governing retrospective studies using anonymized data, written informed consent from parents or legal guardians was not required. All data were handled confidentially and in accordance with institutional data protection policies.

### Statistical analysis

Continuous variables are presented as median [interquartile range, IQR], and categorical variables as number (percentage). Group comparisons for quantitative variables were performed using the Student’s *t-*test or Wilcoxon–Mann–Whitney test, depending on data distribution. Qualitative variables were compared using the Chi-squared or Fisher’s exact test, as appropriate. A *p*-value < 0.05 was considered statistically significant.

Statistical analyses were performed using EasyMedStat software, version 3.42 (www.easymedstat.com, Paris, France).

## Results

### Study population

During the 3-year study period, 870 patients were admitted to the NICU. Among them, 105 (12.1%) died in the NICU and were included in the present analysis, of whom 57% were boys. Most deaths occurred in very preterm infants born before 29 weeks’ GA (*n* = 58, 55.2%), including 24 born at 23–24 weeks’ GA. The second most frequent group (*n* = 27, 25.7%) comprised infants with severe congenital or early-onset conditions, including 9 with polymalformative or genetic syndromes, 7 with complex congenital heart disease, 4 with congenital diaphragmatic hernia, 1 with gastroschisis with antenatal signs of intestinal compromise, 1 with a giant omphalocele, and 3 with rapidly progressive postnatal organ failure (1 neonatal hemochromatosis and 2 major cerebral congenital malformations). All antenatally diagnosed conditions were eligible for termination of pregnancy according to national legislation, but parents elected to continue the pregnancy. The remaining 20 infants (19.0%) died following hypoxic-ischemic encephalopathy.

At the time of death, the median number of organ failures was 2 [IQR 1–3]. Neurological failure was present in 60% of cases, including 24 infants with isolated brain injury. The most frequent other organ failures involved the respiratory (*n* = 62, 59%) and the cardiovascular system (*n* = 58, 55.2%).

A detailed description of the study population according to the reason for admission is provided in Supplementary Table 1.

### Circumstances of death

**Fig. 1:**
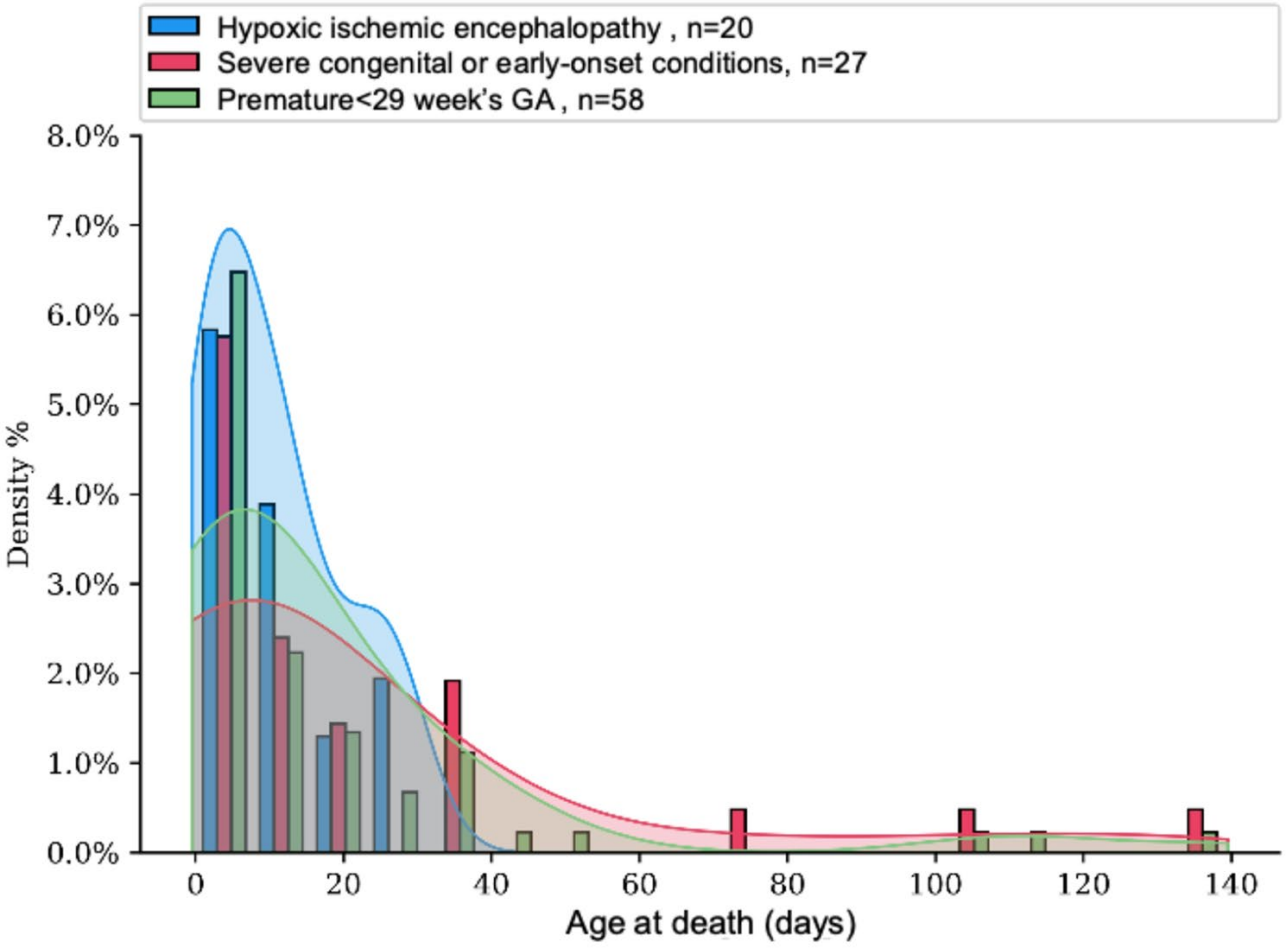
Age at death according to the reason for admission

The median age at death was 8 days [IQR 2–21] (Fig. 1). More than half of the deaths (*n* = 59; 56.2%) occurred during on-call hours.

Overall, 76 infants (72.4%) died following a WWLST decision, corresponding to anticipated deaths. Conversely, 23 deaths (21.9%) were unanticipated, and 6 (5.7%) occurred in the context of brain death, all secondary to severe hypoxic-ischemic encephalopathy. No organ donation was performed.

At the time of death, 95 infants (90.5%) were intubated. The most frequent mode of death was terminal extubation (*n* = 46; 43.8%), followed by therapeutic failure despite ongoing intensive support (*n* = 29; 27.6%). The infant was held in the parents’ arms during end-of-life care in 66 cases (62.8%).

A detailed description of the circumstances of death according to the reason for admission is presented in Table 1.

**Table 1:** Description of the circumstances of death according to the reason for admission

Variable		Total *N*=105	Very premature *N* = 58	Severe congenital conditions *N* = 27	HIE *N* = 20	P*
Age at death (days)		8.0 [2.0; 21.0]	7.5 [2.0; 20.3]	8.0 [2.0; 26.5]	8.0 [2.8; 14.0]	0.95
Death during on-call period		59 (56.2)	33 (56.9)	19 (70.4)	7 (35.0)	0.05
Circumstances of death	Following a WWLST	76 (72.4)	41 (70.7)	22 (81.5)	13 (65.0)	< 0.01
	Unanticipated death	23 (21.9)	17 (29.3)	5 (18.5)	1 (5.0)	-
	Brain death	6 (5.7)	0 (0.0)	0 (0.0)	6 (30.0)	-
Time between complication and death (days)		4.0 [1.0; 12.0]	3.0 [1.0; 8.8]	4.0 [1.5; 7.0]	8.5 [2.8; 13.3]	0.31
Mode of death	Terminal extubation	46 (43.8)	25 (43.1%)	9 (33.33%)	12 (60.0%)	0.15
	Therapeutic failure	29 (27.6)	18 (31.03%)	8 (29.63%)	3 (15.0%)	-
	Unsuccessful CPR	16 (15.2)	11 (18.97%)	4 (14.81%)	1 (5.0%)	-
	Deep continuous sedation	14 (13.3)	4 (6.9%)	6 (22.22%)	4 (20.0%)	-
Parents present		93 (88.6)	52 (89.7)	22 (81.5)	19 (95.0)	0.38

*HIE* hypoxic ischemic encephalopathy, *WWLST* withholding or withdrawing life-sustaining therapies. Values are median [IQR] or *N* (%)

*Comparison between very premature<29 week’s GA, severe congenital or early-onset conditions and hypoxic ischemic encephalopathy groups

### Characteristics of WWLST decisions

Among the 76 deaths following a WWLST decision, 40 (52.6%) involved withdrawal of life-sustaining therapies (Type 3), and 36 (47.4%) involved non-escalation of treatments (Type 1 and 2). Fifteen WWLST decisions (19.7%) were made in emergency situations. In eight cases (10.5%), the request for treatment limitation was initiated by the parents.

The main reasons for WWLST were a poor neurological prognosis (*n* = 39; 51%) and a perceived futility of care (*n* = 34; 45%). Only 30 (39.5%) had three or more organ failures, and most presented with neurological failure (*n* = 51; 67.1%). Isolated renal, digestive, or pulmonary prognoses accounted for one case each.

The median interval between the onset of the major complication and the WWLST decision was 4 days [IQR 1–9], and the median interval between the WWLST decision and death was 2 days [IQR 0–4.3].

Detailed characteristics of WWLST decisions according to the reason for admission are presented in Table 2.

**Table 2:** Description of the WWLST according to the reason for admission

Variable		Total *N*=76	Very premature *N* = 41	Severe congenital conditions *N* = 22	HIE *N* = 13	P*
Time between complication and WWLST (days)		4.0 [1.0; 9.0]	3.0 [1.0; 7.0]	3.0 [0.0; 5.8]	8.0 [6.0; 9.0]	0.03
Main argument supporting WWLST	Neurological outcome	49 (64.5)	22 (53.7)	5 (22.7)	12 (92.3)	<0.01
	Therapeutic futility	34 (44.7)	18 (43.9)	16 (72.7)	0 (0.0)	-
	Others	3 (3.9)	1 (2.4)	1 (4.5)	1 (7.7)	-
Emergency WWLST		15 (19.7)	11 (26.8)	4 (18.2)	0 (0.0)	0.10
WWLST type	1 and 2	36 (47.4)	21 (51.2)	14 (63.6)	1 (7.7)	<0.01
	3	40 (52.6)	20 (48.8)	8 (36.4)	12 (92.3)	-
Time between WWLST and death (days)		2.0 [0.0; 4.3]	1.0 [0.0; 4.0]	2.0 [1.0; 4.0]	4.0 [3.0; 8.0]	0.07

HIE, hypoxic ischemic encephalopathy; WWLST, withholding or withdrawing life-sustaining therapies. Values are median [IQR] or *N* (%)

*Comparison between premature<29 week’s GA, severe congenital or early-onset conditions and hypoxic ischemic encephalopathy groups

### Characteristics of unanticipated deaths

Among the 23 infants who died without a prior WWLST decision (unanticipated deaths), 15 (65.2%) presented with three or more organ failures at the time of death. Almost all (*n* = 22; 95.7%) had cardiovascular failure, and 19 (82.6%) had respiratory failure, while no neurological involvement was identified in 17 cases (73.9%).

The median interval between the last major complication and death was 1 day [IQR 0.5–2]. Fourteen infants experienced cardiac arrest requiring cardiopulmonary resuscitation (CPR), and nine deaths occurred despite refractory multi-organ failure. In most CPR cases (12/14; 86%), the situation was clinically foreseeable and occurred in a context of profound hypoxemia, septic shock with high-dose vasoactive support, or severe metabolic acidosis under renal replacement therapy.

In univariate analysis, the absence of a WWLST decision was associated with shorter NICU stays, shorter intervals between complication and death, and a higher number of concurrent organ failures. Hemodynamic and pulmonary failures were more frequent in this group, whereas neurological involvement was less common. Parents were also less frequently present and less likely to hold their infant at the time of death in situations without WWLST (Table 3).

**Table 3:** Comparison of patient characteristics according to the presence or absence of WLST

Characteristics		WLST (*n* = 76)	No WLST (*n* = 23)	*P*
Boys		44 (57.9)	14 (60.9)	0.99
Broad diagnostic categories	Very premature	41 (54.0)	17 (73.9)	0.17
	Severe congenital conditions	22 (29.0)	5 (21.7)	-
	HIE	13 (17.1)	1 (4.4)	-
Age at death (days)		11.0 [4.0; 26.3]	2.0 [1.0; 7.0]	< 0.01
Interval between complication and death (days)		6.5 [2.8; 16.3]	1.0 [0.5; 2.0]	< 0.01
Death during on-call hours		42 (55.3)	15 (65.2)	0.55
Brain failure		51 (67.1)	6 (26.1)	< 0.01
Hemodynamic failure		35 (46.1)	22 (95.7)	< 0.01
Lung failure		41 (54.0)	19 (82.6)	< 0.01
Kidney failure		20 (26.3)	11 (47.8)	0.07
Number of organ failures	1	26 (34.2)	1 (4.4)	0.02
	2	20 (26.3)	7 (30.4)	-
	≥ 3	30 (39.5)	15 (65.2)	-
Renal replacement therapy		4 (28.6)	5 (45.5)	0.43
Parents during the death	presents	15 (19.7)	12 (52.2)	< 0.01
	in the arms	56 (73.7)	4 (17.4)	-
	not present	5 (6.6)	7 (30.4)	-

*HIE* hypoxic ischemic encephalopathy, *WWLST* withholding or withdrawing life-sustaining therapies. Values are median [IQR] or *N* (%)

## Discussion

This three-year study, including 105 deaths representing 12.1% of all NICU admissions, provides an overview of mortality patterns and EOL circumstances in a tertiary NICU. Most deaths (72%) followed a decision to WWLST, mainly for poor neurological prognosis or therapeutic futility. Conversely, unanticipated deaths were mostly associated with multi-organ failure, minimal neurological involvement, shorter illness trajectories, and limited parental presence.

### Profiles of infants who die in the NICU

Mortality was concentrated in three groups: (i) very preterm infants born before 29 weeks’ GA, (ii) term or near-term infants with hypoxic-ischemic encephalopathy, and (iii) infants with severe congenital or early-onset conditions. Beyond these categories, deaths were rare, underscoring that in high-resource settings, neonatal mortality mainly affects infants with irreversible or extreme conditions.

More than half of the deaths involved very preterm infants, consistent with European and North American data showing that prematurity remains the leading cause of NICU mortality [9, 16]. Outcomes for infants born at 23–24 weeks’ gestation remain poor due to physiological immaturity and the limits of intensive care at the threshold of viability [3, 17, 18]. Survival disparities persist across countries and even between NICUs within the same region, influenced by definitions of viability and local ethical approaches to treatment [17–19].

The second major group comprised infants with congenital malformations or genetic syndromes. Most would have been eligible for medical termination under French law, but parents chose to continue the pregnancy.

### Circumstances and characteristics of EOL care

The overall proportion of deaths following WWLST (72%) was higher than in the national, prospective, population-based cohort study EPIPAGE-2 (≈ 60%), conducted in France in 2011 [19]. The difference stems first from inclusion in our cohort of infants with severe congenital or early-onset conditions. For these newborns, WWLST decisions were anticipated and implemented early (81% of cases). Recent data in our country show that 13% of newborns with incurable fetal disease received perinatal palliative care, with decisions made either during pregnancy or following neonatal assessment [20]. In addition, WWLST was also decided in all but one infant with hypoxic-ischemic encephalopathy treated with active hypothermia, based on a very high perceived risk of severe multiple disabilities. Such redirection of care in this context has been reported by others [21].

EOL care in our unit was characterized by a high rate of terminal extubation and active parental participation, with nearly two-thirds of infants held by their parents during dying. These practices align with European trends emphasizing family-centered and comfort-oriented palliative care [22]. Previous work from our institution found that depression at 5 months after loss was lower when death followed a WWLST process than when it was sudden [7], highlighting the benefit of anticipatory communication and shared decision-making. Although more than half of deaths occurred during on-call hours, this temporal distribution should not be interpreted as reflecting reduced quality of care, as previous studies have shown comparable outcomes during daytime and nighttime in NICUs.

### Unanticipated deaths: opportunities for earlier recognition

Unanticipated deaths (22%) mainly resulted from acute, rapidly progressive multi-organ failure, often involving respiratory and cardiovascular failure. These occurred after shorter NICU stays and shorter intervals between the final complication and death. While some events were unavoidable, most reflected critical deterioration that could potentially have been anticipated, suggesting the need for earlier multidisciplinary discussion with parental involvement. Similar observations in other NICU and PICU settings emphasize improving recognition of poor prognostic trajectories and team communication [7, 8]. Our findings also highlight the importance of facilitating parental presence, even during acute deterioration or resuscitation, as this may profoundly shape families’ experience of their child’s final moments.

Although EOL situations remain unique, structured ethics training and regular case discussions may reduce clinician isolation and foster consistency across units [23–25].

### Perspectives for improving neonatal EOL care

Early identification of infants at risk of dying, combined with structured team meetings and timely parental dialogue, could transform crisis situations into anticipated and supported EOL pathways. Education in neonatal ethics and palliative care should be prioritized for all caregivers. Developing a shared anticipatory culture may enhance the quality, coherence, and humanity of neonatal EOL care.

In this perspective, the antenatal consultation plays a central role, particularly for families expecting an extremely preterm infant. When conducted systematically and grounded in local epidemiological data, antenatal counselling offers parents a clearer understanding of realistic survival and morbidity expectations. Recent work [26] underscores how individualized, evidence-based prenatal discussions can support parental decision-making, foster trust, and facilitate early alignment between families and caregivers regarding goals of care. Integrating such consultations into routine practice may therefore contribute to reducing unanticipated EOL situations and promoting smoother, more coherent care trajectories from the perinatal period onward.

### Study limitations

This study’s retrospective, single-center design limits generalizability and may introduce selection bias. The absence of ECMO and pediatric cardiac surgery in our unit likely influenced case distribution, as some infants requiring such interventions were delivered elsewhere.

Assigning a single principal cause of death remains challenging when multiple factors coexist; to reduce subjectivity, each case was reviewed independently by two investigators using consistent criteria.

Extremely preterm infants, particularly those born before 26 weeks’ gestation, represent a distinct subgroup with specific prognostic and ethical challenges; however, our sample size did not allow meaningful stratified analyses by gestational age or birth weight. International differences in survival of extremely preterm infants reflect not only medical capabilities, but also variability in ethical frameworks, thresholds of viability, and end-of-life decision-making practices, which should be considered when interpreting mortality data.

Finally, the analysis was restricted to deaths within the NICU, excluding delivery-room or post-transfer deaths, which may underestimate the overall burden of perinatal mortality in our population.

## Conclusion

This study provides new insights into who dies and under which circumstances in a tertiary NICU. Identifying groups and risk situations associated with unanticipated deaths may help guide earlier recognition of end-of-life trajectories and strengthen anticipatory care. By highlighting the contrast between anticipated and unanticipated deaths, this study emphasizes that how infants die in the NICU matters—not only clinically, but also in the conditions in which families experience and accompany their child’s death. 

## Supplementary Information

Supplementary Material 1.

## Data Availability

The datasets generated and/or analyzed during the current study are available from the corresponding author upon reasonable request.
