# Supplementary material for: End-of-life circumstances and unanticipated deaths in a neonatal intensive care unit: a retrospective analysis
Source: BMC Palliat Care. 2026 Feb 23;25:74. doi: 10.1186/s12904-026-02014-2 (PMC13036893; doi:10.1186/s12904-026-02014-2)
Supplement: Supplementary file 1 — Supplementary Material 1. [file 12904_2026_2014_MOESM1_ESM.docx]

**Supplementary Table 1. Description of the study population according to the reason for admission**

| **Variable** |  | **Total**  **N=105** | **Premature**  **N = 58** | **Severe congenital conditions**  **N = 27** | **HIE**  **N = 20** | **p-Value** |
| --- | --- | --- | --- | --- | --- | --- |
| 1-min Apgar score |  | 3 [1; 6] | 3 [2; 6] | 4[2; 7.5] | 0 [0; 1] | **<0.01** |
| 5-min Apgar score |  | 6 [3; 8] | 7 [5; 8] | 8 [5; 9] | 1 [0; 4] | **<0.01** |
| 10-min Apgar score |  | 8 [5; 9] | 8 [7; 10] | 8 [5; 10] | 2 [0; 5] | **<0.01** |
| Outborn |  | 31 (29.5) | 11 (19.0) | 7 (25.9) | 13 (65.0) | **<0.01** |
| Renal replacement therapy |  | 9 (8.6) | 3 (5.2) | 3 (11.1) | 3 (15.0) | 0.29 |
| **Number of organ dysfunction** | 1 | 29 (27.6) | 12 (20.7) | 6 (22.2) | 11 (55.0) | 0.05 |
|  | 2 | 28 (26.7) | 17 (29.3) | 8 (29.6) | 3 (15.0) | - |
|  | ≥3 | 48 (45.7) | 29 (50.0) | 13 (48.2) | 6 (30.0) | - |
| Brain dysfunction |  | 63 (60.0) | 33 (56.9) | 10 (37.0) | 20 (100.0) | **<0.01** |
| Lung dysfunction |  | 62 (59.0) | 38 (65.5) | 19 (70.4) | 5 (25.0) | **<0.01** |
| Hemodynamic dysfunction |  | 58 (55.2) | 35 (60.3) | 20 (74.1) | 3 (15.0) | **<0.01** |
| Kidney dysfunction |  | 33 (31.4) | 22 (37.9) | 6 (22.2) | 5 (25.0) | 0.28 |
| Gastrointestinal dysfunction |  | 21 (20.0) | 12 (20.7) | 6 (22.2) | 3 (15.0) | 0.81 |
| Hemostatic dysfunction |  | 11 (10.5) | 5 (8.6) | 2 (7.4) | 4 (20.0) | 0.35 |
| Liver dysfunction |  | 6 (5.7) | 2 (3.5) | 2 (7.4) | 2 (10.0) | 0.46 |

*Legend: HIE, hypoxic ischemic encephalopathy. Values are median [IQR] or N (%).*

**Comparison between premature<29 week’s GA, severe congenital or early-onset conditions and hypoxic ischemic encephalopathy*
